# Clinical significance of wall invasion pattern of subserosa-invasive gallbladder carcinoma

**DOI:** 10.3892/or.2012.1971

**Published:** 2012-08-10

**Authors:** KEN-ICHI OKADA, HIROSHI KIJIMA, TOSHIHIDE IMAIZUMI, KENICHI HIRABAYASHI, MASAHIRO MATSUYAMA, NAOKI YAZAWA, SHOICHI DOWAKI, KOSUKE TOBITA, YASUO OHTANI, MAKIKO TANAKA, SADAKI INOKUCHI, HIROYASU MAKUUCHI

**Affiliations:** 1 Department of Surgery, Tokai University School of Medicine, Isehara, Kanagawa 259-1193; 2 Department of Pathology, Tokai University School of Medicine, Isehara, Kanagawa 259-1193; 3 Department of Critical Care and Emergency Medicine, Tokai University School of Medicine, Isehara, Kanagawa 259-1193; 4 Department of Pathology, Hirosaki University School of Medicine, Hirosaki, Aomori 036-8562, Japan

**Keywords:** gallbladder adenocarcinoma, invasion pattern, subserosal invasion, vascular invasion, prognosis

## Abstract

We have previously classified wall invasion patterns of gallbladder carcinoma (GBC) cases into two groups, i.e., the infiltrative growth type (IG type) and destructive growth type (DG type). The DG type was significantly associated with poor differentiation, aggressive infiltration and decreased postoperative survival in terms of its histological differentiation, lymphatic invasion, venous invasion, lymph node status, neural invasion and mode of subserosal infiltration. In the present study, we analyzed 42 surgically-resected subserosal invasive gallbladder adenocarcinomas, invading the perimuscular connective tissue (pT2). The cumulative 5-year survival rate in the series was 48.7%. Lymphatic invasion (p=0.021), venous invasion (p=0.020), mode of subserosal infiltration (p<0.001), histological differentiation (p=0.030) and biliary infiltration (p=0.007) were noted, respectively, at a significantly higher incidence in more aggressive infiltration or poor differentiation in the DG type. The cumulative 5-year survival rate of curative resection cases was lower in patients with the DG type than in those with the IG type (68.9 versus 20.2%, respectively, p=0.006, log-rank test). On Cox’s proportional hazard regression modeling, the low degree of venous/perineural invasion and IG type of wall invasion pattern were associated with a significant improvement in overall survival. Our data suggest that the wall invasion pattern is an independent predictor of survival in subserosal invasive GBC. Regarding the clinical application of our concept, on the classification of patients with subserosal invasive GBC based on a combination of the wall invasion pattern and lymph node status, the overall survival rate in patients with the DG type and/or N2 metastasis (n=21) was lower than in patients with the IG type and N0, 1 metastasis (n=21) (p=0.0023, log-rank test). The wall invasion pattern could contribute to decision-making concerning curative resection for subserosal invasive GBC.

## Introduction

We have classified the wall invasion pattern of gallbladder carcinoma (GBC) into two groups, i.e., the infiltrative growth type (IG type) and destructive growth type (DG type) (1). The DG type was significantly associated with poor differentiation, aggressive infiltration, and decreased postoperative survival in histological differentiation, lymphatic invasion, venous invasion, lymph node status, neural invasion, and mode of subserosal infiltration. Therefore, the classification of the IG/DG growth pattern is thought to be a useful indicator of the local aggressiveness of GBC. There has been no definition or classification of the wall invasion pattern, and it was defined mainly based on the invasive phenomenon through the muscle layer. We also demonstrated that the wall invasion pattern was correlated with the overall aggressiveness of cancer, i.e., cell proliferation and local aggressiveness of cancer, such as the stromal infiltration of GBC using an immunohistochemical procedure (2). High-grade cell proliferation employing the Ki-67 labeling index (Ki-67 LI) and invasiveness with stromal laminin-5 γ2 chain staining were significantly correlated with an aggressive wall invasion pattern indicating the DG type of GBC. In this study, we analyzed the prognostic value of the wall invasion pattern at a clinically significant depth of tumor invasion, i.e., the subserosal layer.

From the viewpoints of surgical pathology and anatomy, it is sometimes difficult to resect advanced GBC radically (3). There is controversy regarding the surgical indication, such as partial resection of the liver, bile duct resection, and pancreatoduodenectomy for dissecting regional lymph nodes (4–8). Since preoperative evaluation of the tumor spread of subserosa-invasive GBC was difficult, the risk of excessive or inadequate surgery is relatively high. Therefore, it is important to reconsider the surgical procedure to ensure an appropriate operation avoiding oversurgery. Previous studies reported that the presence of lymph node metastases represented the main marker of a poor prognosis (9–12). Intraoperative frozen sections provide important information on lymph node metastasis in surgery. In addition, we propose the clinical use of the wall invasion pattern as a new prognostic predictor which is also available using ordinary hematoxylin-eosin sections. Classification according to the wall invasion pattern and degree of lymph node metastasis would be helpful to re-examine the necessity of an additional surgical procedure in or after the surgery.

## Materials and methods

### Gallbladder tissue specimens

All tissue specimens were obtained on the surgical resection of gallbladder adenocarcinomas at Tokai University Hospital. The subjects were 42 patients (24 men and 18 women; age range 40–93 years; mean age 65.4±10.7 years) with gallbladder tumors invading the subserosal layer, i.e., the perimuscular connective tissue (pT2), at surgery. The stages of GBC were based on the TNM classification. The median postoperative follow-up duration was 852.5 (332.0–2,027.8) days.

### Histological examination

The gallbladder tissue specimens for histological analysis were rapidly fixed in 10% buffered formalin for 24–48 h and routinely embedded in paraffin. Tumor invasion was examined using 4 μm sections stained with hematoxylin and eosin. The degree of venous invasion was classified as: v0, no venous invasion; v1+, minimal venous invasion, i.e., 1 or 2 foci of venous invasion in one histological section; v2+, moderate venous invasion, i.e., 3 or 4 foci; and v3+, marked venous invasion with ≥5 foci. The degree of lymphatic invasion was classified as: ly0, no lymphatic invasion; ly1+, mild lymphatic invasion, ly2+, moderate lymphatic invasion; and ly3+, marked lymphatic invasion. The degree of perineural invasion was classified as: ne0, no perineural invasion; ne1+, mild perineural invasion; ne2+, moderate perineural invasion; and ne3+, marked perineural invasion. The mode of subserosal infiltration was classified into three groups, according to the General Rules for Gastric Cancer Study of the Japanese Gastric Cancer Association (13), i.e., INF alpha α), a cancer nest showing expansive growth and presenting a clear borderline between itself and adipose tissue; INF beta (β), growth and invasive patterns intermediate of those of α and γ; and INF gamma (γ): scirrhous growth, a cancer nest showing invasive growth, while the borderline between the tumor and adipose tissue is unclear. The degree of biliary invasion was classified as: binf0, no invasion to the hepatoduodenal ligament; binf1, uncertain invasion to the hepatoduodenal ligament; binf2, mild invasion to the hepatoduodenal ligament; or binf3, moderate to marked invasion to the hepatoduodenal ligament.

### Definition and histological identification of invasion pattern

**Figure 1:**
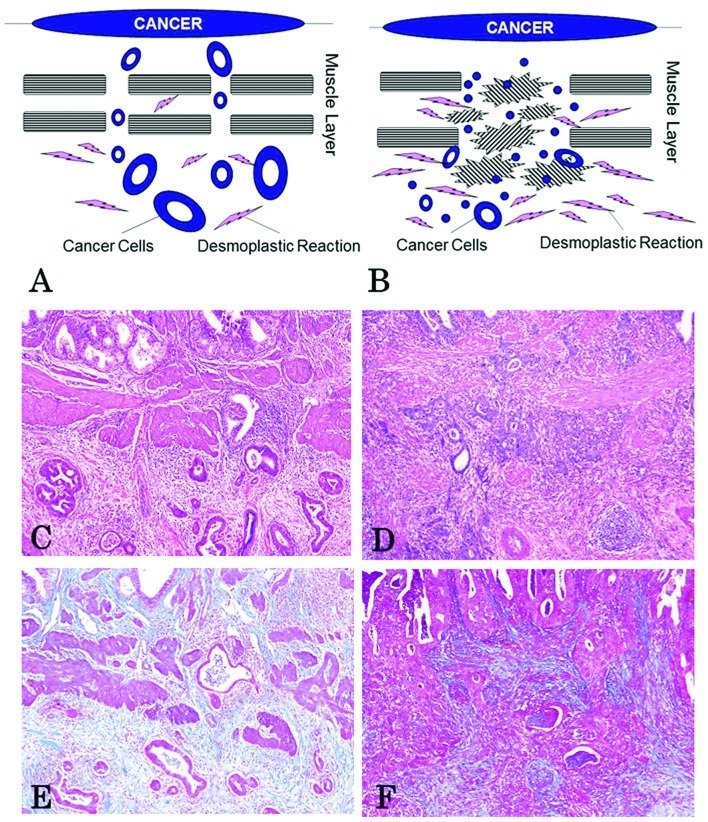
A scheme and microscopic findings of gallbladder carcinoma invasion. IG type (A and C), cancer cells show infiltrative growth in the muscle layer (through the intermuscular space) without muscle layer destruction. DG type (B and D), cancer cells invade the subserosal layer with destruction of the muscle layer. The DG type was accompanied by a stromal desmoplastic reaction with activated fibroblasts and dense collagen fibers, which were aniline blue-positive (E). The IG type revealed a less pronounced reaction of desmoplasia, weakly positive for aniline blue (F). (A and B) Schematic of invasion; (C and D) Hematoxylin-eosin staining; (E and F) Azan staining.

The following terminology was used to define and classify the two patterns of invasion through the muscle layer. Infiltrative growth (IG) type: cancer cells show infiltrative growth in the muscle layer (through the intermuscular space) without muscle layer destruction (Fig. 1A and C) (1). Destructive growth (DG) type: cancer cells show massive growth with destruction of the muscle layer (Fig. 1B and D) (1). The cases showing both DG and IG components were classified as the DG type because aggressive growth patterns were present.

Azan staining was helpful for distinguishing the DG from the IG type of GBC. The DG type usually showed aggressive growth, and included a stromal desmoplastic reaction with activated fibroblasts and dense collagen fibers, which were aniline blue-positive with Azan staining (Fig. 1F). The IG type revealed a lower-level reaction of desmoplasia, which was weakly positive for aniline blue (Fig. 1E).

### Statistical analysis

Descriptive statistical analyses were employed to examine the demographic characteristics of the study population. Data are expressed as means ± SD and medians (25th and 75th percentiles). The baseline characteristics, disease, and pathological variables were compared between patients with the IG and DG types by means of the χ^2^ test for continuous and categorical variables. Univariate analyses (χ^2^ test) were primarily used for selecting variables on the basis of a p<0.05. The significant variables in univariate analyses and clinically significant factors were subjected to Cox’s proportional hazard regression modeling to assess the effect that independent covariates had on the dependent variable of survival. Odds ratios (ORs) and their 95% confidence intervals (CIs) were used to assess the independent contributions of significant factors. A p<0.05 was considered to indicate significance.

Survival times were measured from the date of surgery, and death from all causes (without differentiating between deaths resulting from GBC or other causes) was taken as the outcome. Survival curves were traced with the Kaplan-Meier method, and the comparison of survival curves was carried out using the log-rank test. All analyses were performed using the statistical software package SPSS II (version 11.0; SPSS, Tokyo, Japan).

## Results

**Figure 2:**
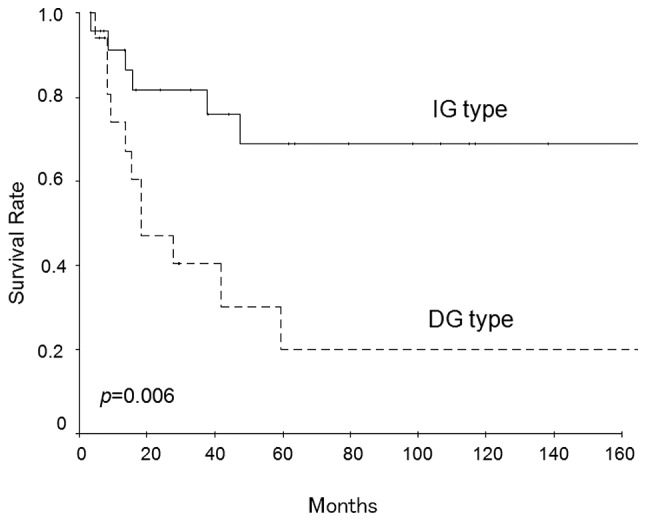
Survival curves of gallbladder cancer patients with subserosal invasion according to the wall invasion pattern. Patients with the DG type (dotted line, n=18) showed a significantly poorer prognosis compared to those with the IG type (solid line, n=24; p=0.006, log-rank test).

Of the 42 subserosa-invasive GBCs (pT2), 24 (57.1%) cases showed the IG type and 18 (42.9%) the DG type. Well to moderately differentiated adenocarcinoma was the most frequent histological type (85.7%). Poorly differentiated adenocarcinoma and other histological types such as signet ring cell carcinoma and adenosquamous cell carcinoma were also observed. We analyzed the relationship between the wall invasion patterns through the muscle layer and clinicopathological features (Table I). Lymphatic invasion (p=0.021), venous invasion (p=0.020), mode of subserosal infiltration (p<0.001), histological differentiation, (p=0.030) and biliary infiltration (p=0.007) were noted, respectively, at a significantly higher incidence in more aggressive infiltration or poor differentiation in the DG type. In addition, cases with the DG type tended to show a higher incidence of neural invasion (p=0.094) and lymph node metastasis (p=0.103). The overall survival rate in the series was 48.7%. Fig. 2 shows the survival curves of patients with each wall invasion pattern. The cumulative 5-year survival rate of curative resection cases was lower in patients with the DG type than in those with the IG type (20.2 versus 68.9%, respectively, p=0.006, log-rank test).

To define the significance of prognostic factors, a Cox’s proportional hazard regression model was designed to assess factors which were significant on univariate analysis. In this model, the low degree of venous/perineural invasion (v0,1+/pn0,1+) and the IG type of wall invasion pattern were associated with a significant improvement in overall survival (Table II).

**Figure 3:**
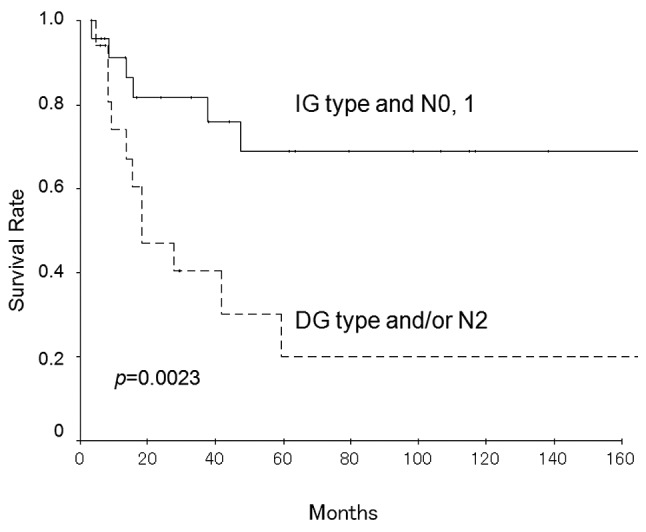
Survival rate of gallbladder cancer patients grouped according to the wall invasion pattern and degree of lymph node metastasis. Patients with the DG type and/or N2 metastasis (dotted line, n=21) showed a shorter survival than those with the IG type and N0, 1 metastasis (solid line, n=21; p=0.0023, log-rank test).

All the cases of subserosa-invasive GBC were categorized into two groups in terms of the wall invasion pattern and lymph node status, and their survival rates were compared using the Kaplan-Meier method and log-rank test. The overall survival rate in patients with the DG type and/or N2 metastasis (n=21) was lower than those with the IG type and N0, 1 metastasis (n=21) (p=0.0023, log-rank test) (Fig. 3).

## Discussion

The radical resection of advanced GBC is sometimes difficult because of frequent lymph node metastasis. In this study, we reviewed 42 surgically resected cases of GBC to clarify the relationship between the wall invasion pattern and clinicopathological features, especially in subserosa-invasive GBC. Lymphatic invasion, venous invasion, distant lymph node metastases, poor differentiation, subserosal scirrhous infiltration (INFγ), and biliary infiltration were more frequently detected in the DG type cases, compared with the IG type cases. This is the first report to describe the relationship between the wall invasion pattern and clinicopathological features of subserosa-invasive GBC.

The layers of the gallbladder wall include the surface epithelium, lamina propria, smooth muscle, perimuscular subserosal connective tissue, and serosa, but they lack the muscularis mucosae and submucosa. The smooth muscle layer consists of loosely arranged bundles of muscle fibers, and is thin compared with other parts of the digestive tract (14,15). Therefore, GBCs can easily invade the subserosal layer through the smooth muscle layer, and show frequent vascular permeation and perineural invasion. Our previous study demonstrated that the wall invasion pattern through the muscle layer is correlated with histological aggressiveness and the survival rate of patients with GBC. The cases in our previous study included not only pT2 GBCs (subserosa-invasive GBCs) but also pT3-4 GBCs together. The bias affected by the depth of tumor invasion in the afore-mentioned clinicopathological relationship could not be excluded. In this study, we clarified that our concept was adequate, according to the greater significance at the same tumor invasion depth, i.e., the subserosal layer is the critical depth both clinically and histologically.

Most GBCs are adenocarcinomas that exhibit the well-differentiated type in the mucosal layer whilst growing laterally and superficially, but display the moderately to poorly differentiated type in the gallbladder wall; therefore, advanced GBCs usually show invasive growth with a desmoplastic reaction, especially from the muscle to subserosal layer (16–22). We propose that the DG type is associated with a more intensive desmoplastic reaction than the IG type; i.e., DG and IG types showed different wall invasion patterns throughout the muscle layers, as well as different subserosal stromal desmoplastic reactions of GBC.

Finally, we discuss the clinical applications of the concept we demonstrated in this study. Surgeons try to perform a potentially curative resection for advanced GBC. However, the true benefits of these radical resections have not been completely established because long-term survivors of advanced GBC are limited. Radical surgery should improve not only survival in early GBC, but should also promote long-term benefits in advanced GBC, which shows high mortality and morbidity. Previous studies have reported the importance of radical lymph node dissection for GBC, and many surgeons have encountered cases in which lymph node dissection improved survival. However, we have encountered cases showing a poor subserosa-invasive GBC prognosis even after radical surgery with lymph node dissection regardless of resection of the other organs, such as the bile duct and liver (6–12). The wall invasion pattern of GBC is easily diagnosed using ordinary hematoxylin-eosin sections, and is applicable to intraoperatively frozen sections. Our data clarified that the wall invasion pattern was an independent predictor of survival in subserosa-invasive GBC, i.e., cases of the DG type and/or N2 metastasis showed a significantly poorer prognosis than those of the IG type and N0, 1 metastasis. Therefore, the wall invasion pattern could contribute to decision-making concerning curative resection for advanced GBC (4,5,23–25).

In conclusion, our study provided evidence to support the concept of a wall invasion pattern in subserosa-invasive GBC. The DG invasion pattern is an indicator of a high malignant potential and indirectly worsens the prognosis of patients with gallbladder adenocarcinoma. To reduce the mortality rate after surgery, we can indicate cases with the IG type and N0, 1 metastasis for radical resection in subserosa-invasive GBC.

## Figures and Tables

**Table I:** The invasion pattern and clinicopathological features of human subserosa-invasive gallbladder cancer.

		Invasion pattern			
				
Clinicopathological features	No. of patients	IG	DG	Rate of DG pattern (%)	p-value χ^2^ test
Histological differentiation					
Well, mod.	36	23	13	36.1	0.030
Poor, other	6	1	5	83.3	
Lymphatic invasion					
ly0, 1+	29	20	9	31.0	0.021
ly2+, 3+	13	4	9	69.2	
Venous invasion					
v0, 1+	27	19	8	29.6	0.020
v2+, 3+	15	5	10	66.7	
Nodal status					
pN0, 1	33	21	12	36.4	0.103
pN2	9	3	6	66.7	
Neural invasion					
ne0, 1+	27	18	9	33.3	0.094
ne2+, 3+	15	6	9	60.0	
Subserosal infiltration					
INFα, β	29	22	7	24.1	<0.001
INFγ	13	2	11	84.6	
Biliary invasion					
binf0, 1	32	22	10	31.3	0.007
binf2, 3	10	2	8	80.0	
Overall	42	24	18	42.9	

IG, infiltrative growth type; DG, destructive growth type; ly, degree of lymphatic invasion; v, venous invasion; ne, neural invasion; binf, biliary invasion. INF α, β and γ, the mode of subserosal infiltration; N, the lymph node status based on the TNM classification. See Materials and methods.

**Table II:** Cox’s proportional hazards model of human subserosa-invasive gallbladder cancer.

Factor	Risk ratio	p-value	95% confidence interval
Sex	0.566	0.280	0.201–1.589
Venous invasion	0.154	0.042	0.025–0.931
Perineural invasion	20.079	0.002	2.959–136.241
Invasion pattern	3.691	0.020	1.232–11.058
